# Soft-matter-based topological vertical cavity surface emitting lasers

**DOI:** 10.1038/s41377-025-02011-9

**Published:** 2026-01-02

**Authors:** Yu Wang, Shiqi Xia, Qun Xie, Donghao Yang, Jingbin Shao, Xinzheng Zhang, Irena Drevensek-Olenik, Qiang Wu, Zhigang Chen, Jingjun Xu

**Affiliations:** 1 The MOE Key Laboratory of Weak-Light Nonlinear Photonics, TEDA Institute of Applied Physics and School of Physics, Nankai University, Tianjin, 300457 China; 2 Collaborative Innovation Center of Extreme Optics, Shanxi University, Taiyuan, 030006 Shanxi China; 3 International Sino-Slovenian Joint Research Center on Liquid Crystal Photonics, Nankai University, Tianjin, 300071 China; 4 Faculty of Mathematics and Physics, University of Ljubljana, and Department of Complex Matter, J. Stefan Institute, SI-1000 Ljubljana, Slovenia

**Keywords:** Nanophotonics and plasmonics, Liquid crystals

## Abstract

Polarized topological vertical cavity surface-emitting lasers (VCSELs) are promising candidates for stable and efficient on-chip light sources, with significant potential for advancing optical storage and communication technologies. However, most semiconductor-based topological lasers rely on intricate fabrication techniques and face limitations in providing the flexibility needed for diverse device applications. By drawing an analogy to two-dimensional Semenov insulators and the quantum valley Hall effect in a synthetic parameter space, we design and realize a one-dimensional optical superlattice using stacked polymerized cholesteric liquid crystal films and Mylar films. Such a one-dimensional optical superlattice is achieved by using films spin-coated with a Pyrromethene 597 solution, thus enabling the demonstration of a structure-flexible, low threshold, and circularly-polarized topological VCSEL. We demonstrate that such a topological VCSEL maintains excellent single-mode operation at low pump power, and its spatial profile aligns closely with that of the pump laser. Thanks to the soft-matter-based metastructure, the topological laser can be “attached” to substrates of various shapes, maintaining desired laser properties and beam steering even after undergoing multiple bends. These characteristics make the demonstrated topological laser ideal for applications in consumer electronics, laser scanning, displays, and photonic wearable devices, where both flexibility and performance are crucial.

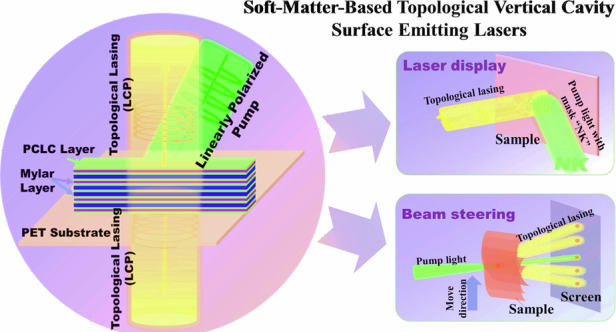

## Abstract (web summary)

A flexible topological vertical-cavity surface-emitting laser (VCSEL) is demonstrated by integrating two one-dimensional optical superlattices composed of structurally distinct polymerized chiral liquid crystals and Mylar films, offering promising prospects for laser display and beam steering applications.

## Introduction

In recent years, the field of topological physics has advanced rapidly, spurring research across a wide range of disciplines including solid-state physics, cold atoms, photonics, acoustics and mechanics^1–6^. After the first theoretical proposal^7^ and pioneering experimental demonstrations^8–10^ in the field of photonics, a variety of topological phenomena have been explored in various photonic systems including plasmonics, metamaterials, coupled resonators, waveguide arrays, polaritonic microcavities and optical systems with synthetic dimensions^3,5,11–16^. These advancements have paved the way for new approaches in implementing nanophotonic devices based on robust topological states. One of the most promising and practical applications of topological photonics lies in the development of topological insulator lasers – devices that leverage the topologically protected boundary states, providing a means to achieve stable and efficient lasing with immunity to disorder and imperfections. Over the past several years, a series of experiments have demonstrated the potential of topological insulator lasers, marking a significant advancement in the application of topological concepts to real-world photonic devices^17–32^.

Driven by the practical applications of integrated photonics, there is a growing need for reliable miniatured on-chip laser sources^33,34^. In particular, vertical cavity surface-emitting lasers (VCSELs) with polarization characteristics have gradually become the core devices for the next generation of optical data storage and optical communications^35–37^, with also a wide range of applications in medical imaging, environmental monitoring, laser scanning, and so on. Recently, topological VCSELs have been proposed and demonstrated, either as a single laser or an arrayed light source, featuring optimal lasing performance such as directionality, low-threshold and high-robustness^26,27^. However, topological VCSELs demonstrated so far are mostly based on semiconductor gain media and solid-state substrates. Due to the increasing demand for laser beam steering technologies and portable laser display devices, a natural question arises: can a topological VCSEL be made shape-flexible, lightweight and chip-compatible, all while maintaining low cost?

On the other hand, soft-matter multifunctional photonic materials with self-supporting properties have significantly advanced the development of wearable devices. These materials, which can be lightweight, flexible, and highly adaptable, have demonstrated superior performance in nonlinear optics and photonics^38–40^. By harnessing the unique properties of soft matter, such as elasticity, deformability, and ease of integration with various substrates, these materials enable the creation of flexible photonic systems that can maintain high performance even when subjected to mechanical deformation. For example, cholesteric liquid crystal (CLC), as a typical soft substance, not only has birefringence and one-dimensional (1D) photonic band gaps, but also has an ordered and controllable molecular arrangement^41,42^. Taking advantages of those properties, topological lasing has recently been realized using a 1D polymer-CLC superlattice, exhibiting properties such as low threshold, tunability, and circular polarization^30^. However, due to the fluidity of the CLC, such an in-plane topological laser cannot sustain high pump intensity. To overcome the fluidity and instability while still retain the optical properties of CLCs, polymerized CLC (PCLC) films have attracted increasing attention. Since PCLCs can easily be processed into thin films, they are ideal for a flexible VCSEL which can be built with a PCLC Fabry-Perot resonator and organic gain material. With appropriate implementation, PCLC thin films can be used for flexible defect-mode lasers which can be attached to substrates of any sizes and shapes^43^. Topological VCSELs, if assembled with such PCLC thin films, can also be shape-flexible with increasing application potentials.

In this work, we demonstrate for the first time to our knowledge a circularly polarized, PCLC-based, topological VCSEL by juxtaposing two 1D optical superlattices with opposite potential offsets. Such a laser design relies on a physical mechanism analogous to the Semenov insulator and the valley quantum Hall effect in a 2D synthetic parametric space. Compared with similar VCSELs demonstrated before, the metastructural superlattices forming the vertical cavity in our VCSEL setting are prepared by stacking commercial Mylar films and PCLC films spin-coated with a Pyrromethene 597 (PM597) solution in ethanol with a negligible thickness, which can be made flexible and do not require complex processes such as lithography, deposition and etching. The lasing threshold is as low as about 0.47 μJ (1.5 MW cm^−2^) and a total lasing slope efficiency is 4.0%. Such a soft-matter-based topological VCSEL may be further developed for potential applications in areas such as artificial intelligence, sensing, and display devices.

## Results

### Construction of a 1D flexible topological VCSEL by use of synthetic parameter space

In condensed matter physics, the combination of the time-reversal and space-inversion symmetries protects the gapless properties of Dirac fermions in graphene. However, by introducing atoms with different masses in the honeycomb lattice and breaking the inversion symmetry, an energy gap can be created while there is no topological edge state within the bandgap. This system, insulating both in the bulk and along its edge, is called a Semenov insulator, as first discussed by Semenov^44,45^. Although Semenov insulators, such as boron nitride (BN) and silicon carbide (SiC) lattice structures, exhibit topologically trivial properties, it is well established that juxtaposing two “mass-inversed” BN lattices with different valley Chern numbers can lead to a topologically protected mode, in a manner similar to the quantum valley Hall effect^46–50^.

According to the Hamiltonian described by the Dirac equation, it can be found that the on-site potential can be equivalent to the mass term of the atom within a unit cell^51^. Recently, Huang et al. designed an acoustic structure based on the discrete spring mass model and modulated the height of the resonator to simulate the disturbed mass^52^. The calculated band structure shows that one interface state can also be generated by juxtaposing two topologically trivial structures with inversion symmetry broken, and the sound wave can be strongly localized at the interface. The similarities between this 1D system and a 2D hexagonal BN lattice make us wonder whether a 1D equally-coupled and site-potential modulated diatomic chain can have similar topological properties with the 2D quantum valley Hall effect.

In general, the dimension of a physical system is determined by its geometry. Constructing a synthetic space by coupling states in an artificial lattice or introducing structural parameters can provide an ingenious way to realize a high-dimensional system^53–55^. Here, we construct a 1D flexible metastructural superlattice with PCLC films and polymer Mylar films. By adjusting the thickness of the Mylar films, the on-site potentials are modulated, similar to the mass modulation in Semenov insulators.

**Fig. 1:**
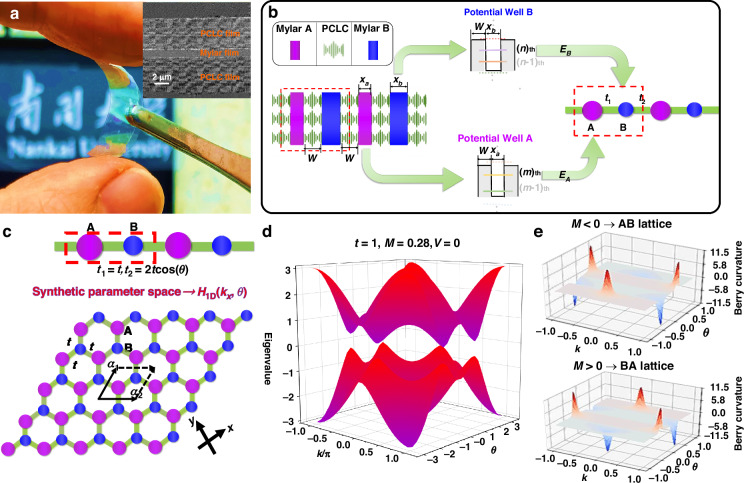
Illustration of the basic principle to construct a 1D flexible optical superlattice and synthetic parameter space. **a** Photography of a free-standing three-layer metastructure consisting of an isotropic Mylar layer (transparent film) sandwiched between two free-standing PCLC layers (selective reflection films). The upper right inset shows the cross-sectional scanning electron microscope (SEM) image of this free-standing structure. **b** Schematic diagram of a 1D AB diatomic chain assembled by two kinds of optical potential wells (Mylar films of different thickness). **c** Introducing coupling coefficient modulation in the 1D AB diatomic chain to construct a synthetic parameter space in analogy with a 2D Semenov insulator. Here, coupling strength *t*_1_ = *t,* *t* ∈ **R**, and *t*_2_ is a variable, ${{{t}}}_{2}=2{{t}}\cos \left({{\theta }}\right),{{\theta }}\in \left[-{\rm{\pi }},{\rm{\pi }}\right]$. **d** Band structure of the coupling-modulated diatomic chain in the *k*-θ synthetic parameter space. **e** Berry curvature of the energy bands below the band gap in the synthetic parameter space for the AB- and BA-type diatomic lattices

Based on the optical quantum-well theory, we first analyze the 1D optical potential well composed of an isotropic polymer layer (transparent film) inserted between two free-standing PCLC layers (selective reflection films), as shown in Fig. 1a. The multilayer films were cut and sprayed with gold powder so as to observe their cross-sectional SEM image, as illustrated in the upright inset. Studies have shown that one or more defect modes can be introduced into the polarization band gap of the CLC by adding isotropic layers and/or pitch jumps within the CLC helix^56^. Here, the PCLC has the characteristics as 1D photonic crystals, and its polarization band gap limits the propagation of light at specific frequencies and polarizations, so it can be regarded as an optical potential barrier. A commercially available isotropic Mylar film (Mylar D, DuPont) is inserted into the PCLC, which serves as a potential well to form an optical quantum well^57,58^. The width of the single potential well is so narrow that the motion of the photons along the direction perpendicular to the well walls exhibits quantized characteristics, and the energies take discrete values in the polarization band gap of the PCLC. Similar to the semiconductor quantum wells, the frequencies of the photon bound states decrease as the well width increases^59–61^. [see Supplementary Note 1 of SI].

For the optical lattice shown in Fig. 1b, different thicknesses of Mylar films cause the change of the coupling energy levels in adjacent potential wells, which in turn change the on-site potentials. Here, we consider only the nearest neighbor coupling, that is, one set of energy levels that can be coupled in the adjacent A and B quantum wells, such as the *m*^th^ energy level in the A potential well and the *n*^th^ energy level in the B potential well. The corresponding energies are defined as *E*_A_ and *E*_B_, which can also be considered as the on-site potentials of A and B sites according to Fig. 1b.

The 1D diatomic chain consists of *N* unit cells, and each unit cell hosts two sites (potential wells A and B). Under the tight-binding approximation where only nearest-neighbor interactions are retained, the Hamiltonian of this 1D diatomic chain can be expressed as:

1 $${{\rm{H}}}_{1{\rm{D}}}=\left[\begin{array}{cc}{{V}}-{{M}} & {{{t}}}_{1}+{{{t}}}_{2}{{\rm{e}}}^{{\rm{i}}{{k}}}\\ {{{t}}}_{1}+{{{t}}}_{2}{{\rm{e}}}^{-{\rm{i}}{{k}}}{} & {{V}}+{{M}}\end{array}\right]$$

where *t*_1_ and *t*_2_ represent the coupling term between the nearest neighbor potential wells. *V* ± *M* represents the potentials on A and B sites. The *V* (average potential of A and B sites) is a constant term to the energy. *M* describes the difference in potentials between A and B sites. Notably, this 1D diatomic chain has spatial inversion symmetry only when the potentials of A and B sites are equal, namely *M* = 0. As a result, *M* is often called the inversion symmetry breaking mass, or the Semenov mass^62^. To explore the topological origin of the 1D onsite-potential modulated diatomic chain, we set *t*_1_ = *t*, *t*_2_ = 2*t* cos(*θ*), in which *θ*=[−π, π] and *t* ∈ **R**, so that we form a synthetic parameter space. The term 2*t* cos (*θ*) can be rewritten as $2{{t}}\cos \left(\theta \right)={{t}}({e}^{{\rm{i}}\theta }+{e}^{-{\rm{i}}\theta })$, and Eq. (1) transforms into:

2 $${{\rm{H}}}_{2{\rm{D}}}\left(k,\theta \right)=\left[\begin{array}{cc}V-M & {{t}}+{{t}}({e}^{{\rm{i}}\left({k}_{x}a+{k}_{y}b\right)}+{e}^{{\rm{i}}\left({k}_{x}a-{k}_{y}b\right)})\\ {{t}}+{{t}}({e}^{-{\rm{i}}\left({k}_{x}a+{k}_{y}b\right)}+{e}^{-{\rm{i}}\left({k}_{x}a-{k}_{y}b\right)}) & V+M\end{array}\right]$$

where some substitutions are used (e.g., $k=$*k*_*x*_$a$ and $\theta =$*k*_*y*_$b$). ${{\boldsymbol{a}}}_{{\bf{1}}}={{a}}\vec{{{x}}}+{{b}}\vec{{{y}}}$ and ${{\boldsymbol{a}}}_{{\bf{2}}}={{a}}\vec{{{x}}}-{{b}}\vec{{{y}}}$ are the basis vectors in the real space, in which *a* and *b* depend on the lattice constants. Here, *θ* acts as a synthetic parameter, enabling us to map this 1D model onto a 2D inversion-symmetry-breaking Semenov insulator, as illustrated in Fig. 1c. The topological properties of this equally-coupled and on-site-potential-modulated diatomic chain can be understood by analyzing a specific case (*k*_*y*_${b=}$π ⁄ 3) of the 2D Semenov insulator. From Eq. (2), the band structure of the 1D onsite-potential and coupling-modulated diatomic chain in the *k*-*θ* synthetic parametric space can be calculated as shown in Fig. 1d. A band gap opens at Dirac points, showing typical characteristics of the Semenov insulators.

**Fig. 2:**
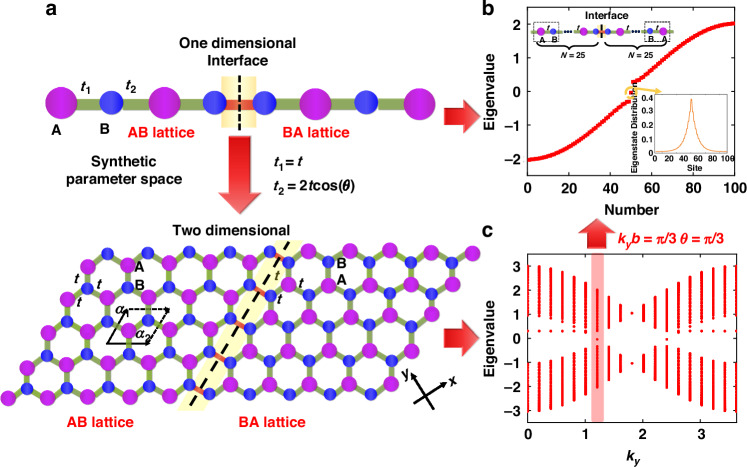
Topological interface states (TISs) generated by juxtaposing AB and BA lattices with different valley Chern numbers. **a** Schematic diagram of the corresponding relationship between the 1D and 2D juxtaposed structures. The yellow areas and dashed lines mark the location of the interface, and both structures on either side of the interface break the inversion symmetry. **b** Eigenvalues and TIS distribution in the 1D equally-coupled diatomic chain. N represents the number of the unit cells at either side of the interface. A topologically protected edge mode is localized at the interface of the AB and BA lattices. **c** Dispersion relationship of the 2D structure juxtaposed by AB and BA lattices with different valley Chern numbers. Here, we set *t* = 1, *b* = $\sqrt{3}$/2 and *M* = 0.28 in our calculation. The red area marks the special points satisfying *k*_*y*_*b* = π/3, namely the cross sections *θ* = π/3 in the synthetic parameter space. In this case, the coupling strength *t*_1_ is equal to *t*_2_ (*t*_1_ = *t*_2_ > 0) in the 1D diatomic chain structure

As Fig. 1e shows, the calculated Berry curvature^63,64^ of the energy bands below the band gap in the synthetic parameter space for the AB lattices has an opposite sign as compared with that for the BA lattices. From *M* < 0 to *M* > 0, A and B atoms are exchanged in the lattices. The valley Chern numbers can be computed by integrating the Berry curvature, which are approximately equal to – 0.4 and + 0.4 for AB and BA lattices at *K’* point, respectively. Here, the Chern numbers deviate from the ideal value of ± 0.5, possibly due to that the extension of the Berry curvature causes the reduction of the integral for the Chern number^46,48^. Consequently, AB and BA lattices have different valley Chern numbers, which leads to a topologically protected interface mode as shown in Fig. 2a, analogous to the quantum valley Hall effect^65^.

Similar to the discussion in the 2D BN lattice structure and other Semenov insulators, we pay attention to the difference in topological properties caused by the Semenov mass *M*, so we only consider the case of *t*_1_ = *t*_2_ > 0 in Fig. 2a. From the dispersion relationship shown in Fig. 2c, we can find the topological interfaces states (TISs) inside the band gap, which are caused by juxtaposing two Semenov insulators with different valley Chern numbers. At the special point *k*_*y*_*b* = π/3, the eigenvalue of the TIS is located exactly in the center of the band gap. According to the corresponding relationship in Eq. (2), this particular point also corresponds to the cross section *θ* = π/3 of the synthetic parameter space. Thus, we can construct a simple 1D equally-coupled diatomic chain with only on-site potential modulated. For example, when we place two equally-coupled diatomic chains (AB and BA) side by side, each with 25 unit cells, a TIS appears right in the center of the band gap, localized at the interface as shown in Fig. 2b.

Further, our optical superlattice is a multi-energy level system. Energy levels with the same energy may correspond to different orders in different potential wells, as shown in Supplementary Table S1 of the SI. It can be found that there are a group of Mylar films with different thicknesses corresponding to the same wavelength of 574 nm, that is, potential wells with different widths can have different energy levels corresponding to the same energy. The quantum size effect and multi-level characteristics of quantum wells allow a bandgap closure to occur even when different Mylar films are used as A, B sites, as shown in the Supplementary Note 2 of the SI.

### Topological interface states in a soft-matter based VCSEL

**Fig. 3:**
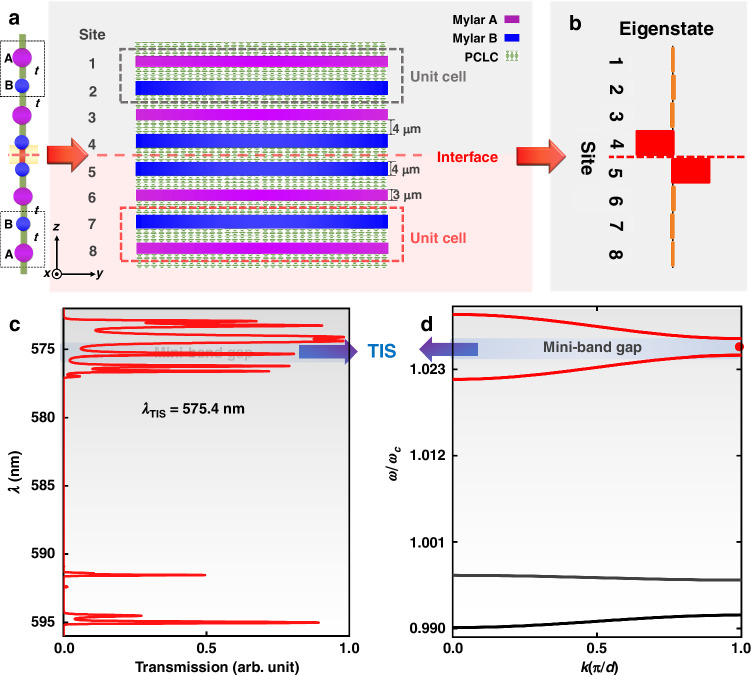
Calculated TIS in the metastructural superlattice used for realizing topological VCSELs. **a** Illustration of the 1D topological VCSEL structure composed of two distinct sub-superlattices with different topological properties, which is equivalent to a 1D equally-coupled diatomic chain with different on-site potentials. **b** Eigenstate distribution of the TIS. **c** Transmission spectrum and **d** mini-band structure of the topological optical superlattice, where the arrows mark the in-gap TIS

The above illustration and analysis reveal the topological origin of the 1D equally-coupled diatomic chain model with only on-site potential modulated. Next, we provide further theoretical calculation and experimental measurement, and finally achieve a topological VCSEL based on this optical superlattice model. Here, we choose “3 μm” -thick Mylar films (as A sites) and “4 μm” -thick Mylar films (as B sites) to construct AB lattice and BA lattice (Fig. 3a). The on-site potentials in the sub-structures of gray and red areas satisfy *E*_A_ > *E*_B_ (*M* < 0) and *E*_A_ < *E*_B_ (*M* > 0), respectively. The topological phase transition process of the AB and BA equally-coupled diatomic chains can also be verified according to the symmetry of the electric field distributions of the eigenstates above and below their common band gap at the edge of the first Brillouin region, as shown in the Supplementary Note 3 of the SI. Here, potential wells A and B have similar energies near the wavelength of 575 nm, corresponding to the *m*^th^ and *n*^th^ energy levels, respectively. Due to the tunneling and coupling between potential wells A and B, the original degenerate energy levels split, and a series of splitting resonance peaks appear in the transmission spectrum^59–61^. In turn, mini-bands are formed and mini-band gaps are generated. This phenomenon can also be analyzed according to the tight binding method^66^.

By juxtaposing two topologically unequal AB lattice and BA lattice with different valley Chern numbers, a TIS which is highly localized at the interface can be generated, as shown in Fig. 3b. It is clear that this result is in agreement with the theoretical calculation of the 1D equally-coupled diatomic chain model shown in Fig. 2a, b. From the calculated transmission spectrum and the mini-band structure of the superlattice, it can be found that the TIS with the wavelength of 575.4 nm originates from one of the bulk states and evolves into the mini-band gap, that is, one bulk state turns into the TIS, as indicated by the arrows in Fig. 3c, d. In addition, because the energy levels *E*_*m*−1_ and *E*_*n*−1_ inside the two potential wells A and B have a large energy difference, light between the nearest potential wells cannot couple. As a result, we cannot find any TIS within 590–598 nm.

In order to experimentally verify the correctness of the theoretical calculation, the metastructural superlattice shown in Fig. 3a was prepared by using Mylar films stacked with PCLC films. The detailed preparation process is described and shown in the next section and Materials and Methods. The transmission spectra of the AB lattice structure and the superlattice after juxtaposition were tested as shown in the Supplementary Note 4 of the SI. A mini-bandgap can be clearly observed in the transmission spectrum of the AB lattice. A TIS with wavelength of 575.4 nm was observed in the mini-band gap of the juxtaposed structure.

From the perspective of application, the most valuable advantage of the TIS should be its robustness against disorder and defect, which has been discussed in detail based on the SSH model with chiral symmetry protection^67^. In addition, some studies have demonstrated that localization of the topological state can be maintained even if the chiral symmetry is broken by directly perturbing the on-site potentials^68^. From the calculation results presented in Supplementary Note 5 of the SI, we can find that the TIS basically keeps its corresponding eigen-wavelength unchanged until the disturbance factor *γ* is equal to about 0.25. At the same time, the strong localization of the TIS is not affected by the degree of disorder. However, the bulk states start to change once the disorder is introduced. Therefore, compared with bulk states, the TIS is more robust against the perturbation or disturbance on the on-site potentials.

### Lasing characteristics of a soft-matter-based topological VCSEL

**Fig. 4:**
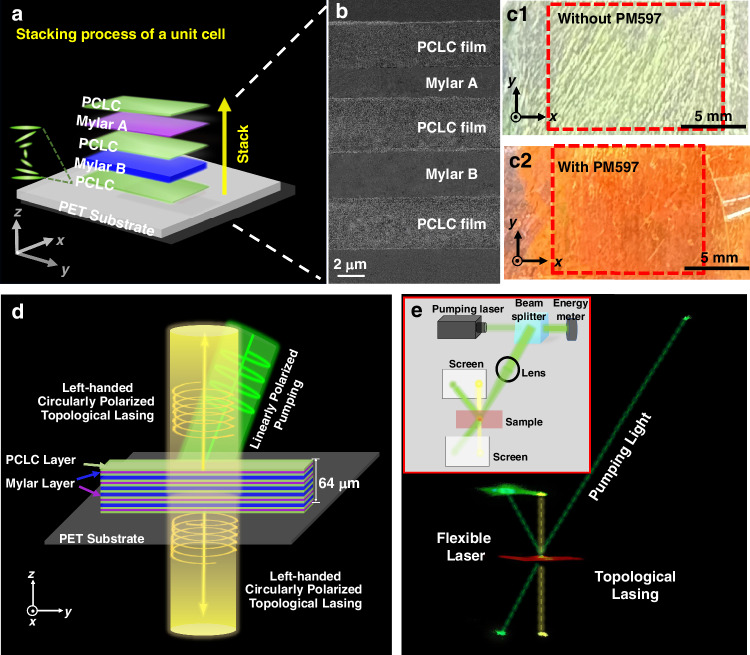
Laser emission from the soft-matter-based topological VCSEL. **a** Schematic diagram of one unit cell with five stacked layers. **b** Cross-sectional SEM image of one unit cell of the topological VCSEL. **c** Pictures of the optical superlattices consisting of 17 layers without (c1) and with (c2) the dye PM597. The areas marked by the red dashed lines are the effective regions of the samples. **d** Illustration diagram of dual-side lasing when pumped by linearly polarized light. The thin film is parallel to the *x-y* plane, and lasing emissions are along the *z* and -*z* directions. **e** A photograph of actual output lasing patterns from two sides of the sample. Due to the fluorescent dye PM597, the film appears purplish red. Two yellow light spots at the direction perpendicular to the sample represent the left-handed circularly polarized TIS laser emission, while the green light spots represent the pump light. The inset shows the corresponding experimental setup

In order to experimentally realize a topological VCSEL, we prepare the soft-matter-based metastructural superlattice cavity by vertically stacking left-handed PCLC films and commercial Mylar films, without any complex lithography or deposition technique. The stacking diagram and the cross-sectional SEM image of one unit cell are shown in Fig. 4a, b. It consists of two Mylar films of different thicknesses (3 μm and 4 μm) and three PCLC films of the same thickness (4 μm). As can be seen from the Fig. 4b, layers are stacked closely and remain flat. By spin-coating a gain medium (Pyrromethene 597 - PM597, solution 1 wt.% in ethanol) with a negligible thickness on each PCLC film, a flexible, free-standing, circularly polarized topological VCSEL can be achieved. Figure 4c1, c2 shows the photographs of two samples with and without PM597, respectively, consisting of 17-layer films on a flexible PET substrate according to the metastructural superlattice shown in Fig. 4d, which is also consistent with the theoretical model in Fig. 3a. The areas framed by the red dashed lines are the effective regions. Here, we used a tape to fix the sample on the substrate at its edges for the purpose of experimental testing. In fact, one can use highly transparent double-faced adhesive tape to attach the film sample virtually to any substrate.

In the experiments, we use a frequency-doubled ${\rm{Q}}$-switched Nd:YAG laser (SLIII-10, Continuum) with an output wavelength of 532 nm, a repetition rate at 10.0 Hz, and a pulse duration of 4.0 ns to excite the sample. Left-handed circularly polarized topological lasing at 575.4 nm is observed from this soft-matter-based topological VCSEL, as shown in Fig. 4d, where two yellow laser beams are illustrated in opposite directions perpendicular to the surface. This wavelength is consistent with that of the TIS in the transmission spectrum calculated theoretically and experimentally (Fig. 3c and Supplementary Fig. S4). A photograph of the actual lasing beam output is shown in Fig. 4e, where the inset shows the specific experimental setup. From this photograph, one can see the lasing and pump light spots on the screens, mirror and sample due to scattering. In order to clearly show the paths of the pump beam and the topological lasing beam, dotted lines are added in Fig. 4e.

**Fig. 5:**
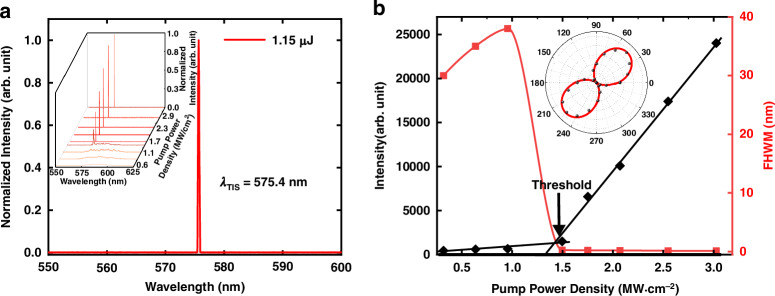
Experimentally measured emission spectra and lasing characteristics of the topological VCSEL. **a** Emission spectrum obtained from the superlattice pumped at 1.15 μJ. The inset shows the emission spectra at different pump energies. **b** Plot of the peak intensity and FWHM of the TIS emission as a function of the pump power density. The laser threshold indicated by an arrow in the figure is 1.5 MW∙cm^−2^. The inset shows the intensity of the quarter-waveplate-transformed laser radiation as a function of the polarization angle in polar coordinates. The polar angle stands for the transmission angle of the polarizer, and the radius stands for the transmittance

We further examine the topological lasing threshold and polarization characteristics through the experimental setup as described in Materials and Methods. In order to improve the spectral characterization and eliminate influence from fluorescent background, we placed the objective lens 2.0 cm behind the sample. Figure 5a shows the single-mode TIS lasing with a peak wavelength of 575.4 nm at a pump energy of 1.15 μJ, and the inset shows the emission spectra under different pump power densities. From Fig. 5b, it can be found that when the pumping energy exceeds 0.47 μJ (1.5 MW∙cm^−2^), the full width at half maximum (FWHM) of the emission peak is obviously narrowed and finally reaches 0.13 nm, indicating the lasing behavior. We also measured the beam divergence angle of the laser by using a beam profile analyzer testing the intensity distribution of laser spots. According to the measurements shown in the Supplementary Note 6 of the SI, the divergence angle of the lasing beam decreases to 3° after the threshold, confirming that our topological VCSEL achieves directional emission. In addition, as the pump energy is larger than 4.4 μJ, other defect states in the superlattice will also be excited. Our experimental results show that the threshold of the TIS lasing is significantly lower than that required to excite those other defect states, because the TIS in the superlattice has the highest photonic density of state. In addition, we also conducted theoretical simulations of our topological VCSEL (see Supplementary Note 7 of the SI). The wavelength of the topological lasing obtained in the calculated emission spectrum has a good correspondence with the experimental result. Meanwhile, the calculated spatial distribution shows that TIS is mainly localized at the interface, which is also in good agreement with the results calculated through the tight-binding model (Figs. 2b and 3b). A quartz quarter-wave plate with its slow axis parallel to the vertical axis and a polarizer are used to examine the polarization characteristics of the TIS lasing. As shown in the inset of Fig. 5b, when the polarizer is rotated to 45° and 225° relative to the horizontal axis, the transmitted intensity is maximum, while the lasing is completely wiped out at 135° and 315°, indicating that the TIS lasing emitted from the topological VCSEL has a good left-handed circular polarization characteristic. Besides, its lasing slope efficiency reaches 4.0%, and its total lasing conversion efficiency (the ratio of lasing pulse energy to pump pulse energy) reaches 3.8% under the pump energy of 2.0 μJ.

### Two application examples of the flexible topological VCSEL

**Fig. 6:**
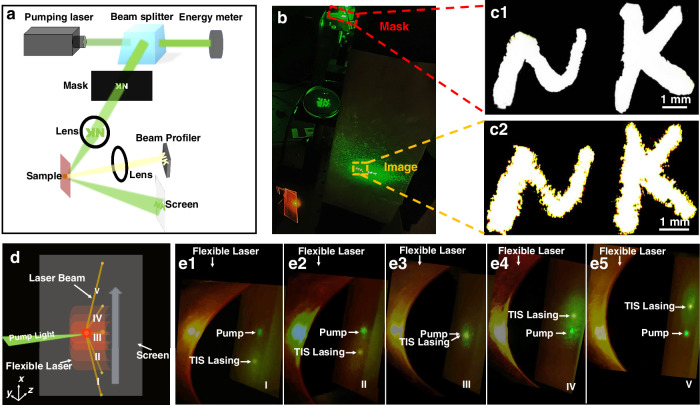
Demonstration of possible applications with the soft-matter-based topological VCSEL. **a** Experimental setup and **b** photograph demonstrating a topological VCSEL excited by a pump beam passing through a photomask containing the letters ‘NK’. **c** Photographs of the photomask (c1) and the topological lasing pattern (c2). The black area of the mask is opaque, while the white area of ‘NK’ is transparent. **d** Illustration diagram of the laser beam steering caused by shifting the curved VCSEL sample. **e**1–5 Experimental photographs taken when the sample is moved upward, and thus the laser illuminates at different positions on the screen (I–V)

Our topological VCSEL not only maintains good single longitudinal mode emission under low pump intensity, but it also carries the spatial information of the pump light, i.e., its spatial profile is determined by the pump light. To illustrate this, a photomask engraved with two letters ‘NK’ is inserted into the pump light path and imaged on the sample by a lens with a focal length of 85 mm. The topological lasing pattern is monitored by a laser beam profiler (SP620U, Ophir-Spiricon) through another lens, as shown in Fig. 6a. As illustrated in Fig. 6b, the pump beam reflected by the sample and the lasing beam can both be displayed on a screen. Figure 6c2 clearly shows the transverse profile of the topological laser, which corresponds well to the photomask shown in Fig. 6c1. Therefore, our topological VCSEL has a great potential to be used in optical display devices. Compared with the traditional optical display devices, it exhibits excellent circular polarization properties, which offers enhanced contrast and improved viewing comfort - features particularly appealing for 3D displays. Importantly, since the helical axis of the PCLC remains perpendicular to the film surface, lasing performance can be maintained during bending. However, there exist several limitations too. For instance, the lasing wavelength cannot be flexibly tuned as in other liquid crystal display devices, the fidelity of far-field images and the laser conversion efficiency are still relatively low, requiring further optimization to meet the demands for practical applications.

Finally, to demonstrate the flexibility of the topological VCSEL, we design an experiment in which the laser beam can be diverted and redirected by moving a curved VCSEL film (see Fig. 1a). As illustrated in Fig. 6d, the sample is bent into a cylindrical surface by fixing it on a cylinder, and moved from bottom to top. As a result, the pump beam of the Nd:YAG laser illuminates different regions of the cylindrical film, so that five different locations on the film are pumped subsequently, labeled as I-V. The deformation of the film causes the helical axes of the PCLCs along different normal directions of the cylindrical surface, which in turn moves the outgoing lasing spots to fall on the screen at different locations. As shown in Fig. 6e1–5, the corresponding TIS lasing spots (yellow spots) and pump lasing spots (green spots) can be seen on the screen, showing the beam steering phenomenon with the flexible topological VCSEL. Adjusting the radius of curvature of the VCSEL film can further control the beam steering degree. This property means that the topological VCSEL can lase in a range of directions without rotating the laser device. Notably, the topological VCSEL is thermally stable and retains its original lasing characteristics once re-pumped even after a long period of time, as shown in the Supplementary Note 8 of the SI. These characteristics of the laser device are particularly attractive for potential wearable photonic technologies and compatible platforms.

## Discussion

We have successfully designed and demonstrated soft-matter-based optical superlattices by integrating PCLC films with different Mylar films, achieving a circularly polarized flexible topological VCSEL in the visible wavelength region. Exploiting the quantum size effect of the equivalent optical quantum wells in the superlattices, we can precisely adjust the energy levels and their spacing by varying the well width, thereby modifying the on-site potentials in the superlattices. Utilizing the synthetic parametric space, we have analyzed the formation of the TIS in our 1D optical superlattice, which is similar to the TIS generated by juxtaposing two Semenov insulators with opposite Semenov mass terms (for example, the hexagonal BN). After introducing the gain medium into the superlattice, a single longitudinal mode TIS lasing can be observed under pulsed laser pumping, which preserves the transverse profile of the pump light. Notably, this topological VCSEL also exhibits a higher laser conversion efficiency and slope efficiency compared to the existing flexible CLC lasers. We have also demonstrated the capability of steering the lasing beam by bending the laser device. This soft-matter-based topological VCSEL features exceptionally low production costs without complex processing technology, and it can be easily integrated onto any substrate. We envision the soft-matter-based topological VCSELs could also be implemented in 2D settings, such as for topological vortex lasers based on twisted moiré lattices^69^. Consequently, the development of such soft-matter-based topological VCSELs holds significant potential for promoting the practical applications of next-generation topological photonic devices.

## Materials and methods

### Fabrication of a soft-matter-based topological VCSEL

**Fig. 7:**
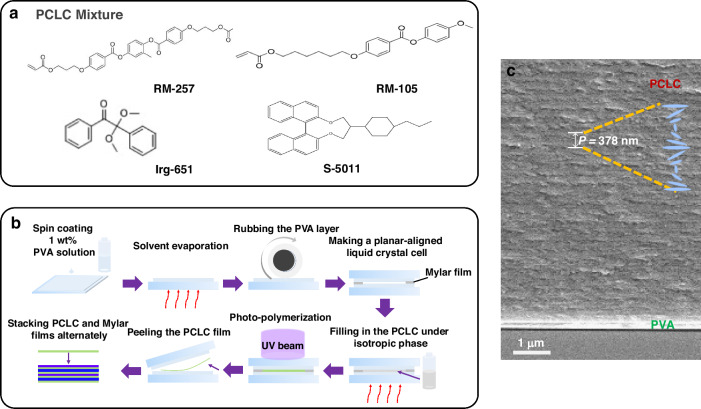
Preparation process of the soft-matter-based topological VCSEL. **a** Structures of molecules used for making PCLC films. **b** Schematic diagram of the fabrication process of the topological VCSEL. **c** Cross-sectional SEM image of the PCLC film. The PCLC has a periodic structure along *z* direction with a pitch of *P*

In order to achieve laser emission from the vertical cavity surface, the helical axis of the PCLC was designed to be perpendicular to the substrate, i.e., along the *z* direction. Thus, we prepared anti-parallel plane-oriented liquid crystal cells by friction orientation technology, and the orientation of liquid crystal molecules was realized by rubbed polyvinyl alcohol (PVA, Sigma-Aldrich) alignment layers. Here, the polymerizable liquid crystal mixture contained 73 wt.% RM257 (JCOPTIX) and 24.5 wt.% RM105 (JCOPTIX), which together formed a basic nematic mixture. Then, 2.05 wt.% left-handed chiral dopant S5011 with a high torque coefficient and 0.45 wt.% photo-initiator Irgacure-651 (BASF) were added as shown in Fig. 7a. These mixtures were filled into lab-made planar LC cells. The thickness of the LC cells was controlled by a 4 μm-thick Mylar film as the spacer. In order to achieve a uniform helical alignment of the PCLC, the sample was cooled from the isotropic phase slowly, and a clear photonic band gap of the PCLC from 560 nm to 604 nm can be seen from its transmission spectrum. A UV curable source (HTLD-4, Height-LED Opto-electronic Tech Co., Ltd) with a wavelength of 365 nm and an intensity of 100 mW∙cm^−2^) was used to irradiate the sample from a distance of 20 cm. In order to ensure uniform curing, both sides of each LC cell were irradiated for 5 min. Then, the glass substrates of the LC cell were torn apart, and the independent PCLC film was peeled off with a sharp tweezer, as shown in Fig. 7b. Figure 7c is a cross-sectional SEM image of one detached PCLC film. Here, PCLC shows a clearly periodic structure along the *z* direction. In addition, among commonly used fluorescent dyes, Pyrromethene 597 (PM597) is the optimal choice for our gain medium (see discussion in Supplementary Note 9 of the SI). Then, a dye-doped flexible topological VCSEL was constructed by alternately stacking the detached PCLC films with commercial Mylar films.

### Experimental setup for lasing measurements

In the experiment, we examined the topological lasing threshold and polarization characteristics of the PM597 dye-doped topological VCSEL. The frequency-doubled Q-switched Nd: YAG laser (SLIII-10, Continuum) was used to excite the sample, with a wavelength of 532 nm, a repetition rate at 10.0 Hz, and a pulse duration of 4.0 ns. The pump light was divided into two beams by a beam splitter. An energy meter (LabMax-Top, Coherent Inc.) was used to monitor the pump energy in the transmitted direction. A lens with a focal length of 85 mm was used to focus the beam in the reflected direction onto the sample. To avoid the influence of the pump laser and protect the spectrometer, a 532 nm notch filter was placed behind the sample. The TIS lasing was collected by a 10× objective lens. The light passing through another objective was coupled to an optical fiber of a high-resolution spectrometer (SP2358, Princeton Instruments, resolution: 0.05 nm). Here, we also measured the fluorescence spectrum of 1.0 wt.% PM597 solution in ethanol, whose fluorescent band ranges from 535 nm to 615 nm, as shown in Supplementary Fig. S9 of the SI.

## Supplementary information

Supporting Information

## Data Availability

The data that supports the plots within this paper and other finding of this study are available from the corresponding author upon reasonable request.

## References

[1] Qi, X. L. & Zhang, S. C. Topological insulators and superconductors. *Rev. Mod. Phys.* **83**, 1057–1110 (2011).

[2] Cooper, N. R., Dalibard, J. & Spielman, I. B. Topological bands for ultracold atoms. *Rev. Mod. Phys.* **91**, 015005 (2019). 10.1103/revmodphys.91.015005 PMC7079706 32189812

[3] Ozawa, T. et al. Topological photonics. *Rev. Mod. Phys.* **91**, 015006 (2019).

[4] Von Klitzing, K. et al. 40 years of the quantum Hall effect. *Nat. Rev. Phys.* **2**, 397–401 (2020).

[5] Zhang, X. J. et al. A second wave of topological phenomena in photonics and acoustics. *Nature* **618**, 687–697 (2023). 37344649 10.1038/s41586-023-06163-9

[6] Shah, T. et al. *Colloquium*: topologically protected transport in engineered mechanical systems. *Rev. Mod. Phys.* **96**, 021002 (2024).

[7] Haldane, F. D. M. & Raghu, S. Possible realization of directional optical waveguides in photonic crystals with broken time-reversal symmetry. *Phys. Rev. Lett.* **100**, 013904 (2008). 18232766 10.1103/PhysRevLett.100.013904

[8] Wang, Z. et al. Observation of unidirectional backscattering-immune topological electromagnetic states. *Nature* **461**, 772–775 (2009). 19812669 10.1038/nature08293

[9] Rechtsman, M. C. et al. Photonic Floquet topological insulators. *Nature* **496**, 196–200 (2013). 23579677 10.1038/nature12066

[10] Hafezi, M. et al. Imaging topological edge states in silicon photonics. *Nat. Photonics* **7**, 1001–1005 (2013).

[11] Price, H. et al. Roadmap on topological photonics. *J. Phys. Photonics* **4**, 032501 (2022).

[12] Ota, Y. et al. Active topological photonics. *Nanophotonics* **9**, 547–567 (2020).

[13] Smirnova, D. et al. Nonlinear topological photonics. *Appl. Phys. Rev.* **7**, 021306 (2020).

[14] Segev, M. & Bandres, M. A. Topological photonics: where do we go from here?. *Nanophotonics* **10**, 425–434 (2020).

[15] Chen, Z. G. & Segev, M. Highlighting photonics: looking into the next decade. *eLight* **1**, 2 (2021).

[16] Khanikaev, A. B. & Alù, A. Topological photonics: robustness and beyond. *Nat. Commun.* **15**, 931 (2024). 38296991 10.1038/s41467-024-45194-2 PMC10831052

[17] St-Jean, P. et al. Lasing in topological edge states of a one-dimensional lattice. *Nat. Photonics* **11**, 651–656 (2017).

[18] Bahari, B. et al. Nonreciprocal lasing in topological cavities of arbitrary geometries. *Science* **358**, 636–640 (2017). 29025992 10.1126/science.aao4551

[19] Harari, G. et al. Topological insulator laser: theory. *Science* **359**, eaar4003 (2018). 29420260 10.1126/science.aar4003

[20] Bandres, M. A. et al. Topological insulator laser: experiments. *Science* **359**, eaar4005 (2018). 29420263 10.1126/science.aar4005

[21] Zhao, H. et al. Topological hybrid silicon microlasers. *Nat. Commun.* **9**, 981 (2018). 29515127 10.1038/s41467-018-03434-2 PMC5841408

[22] Parto, M. et al. Edge-mode lasing in 1D topological active arrays. *Phys. Rev. Lett.* **120**, 113901 (2018). 29601765 10.1103/PhysRevLett.120.113901

[23] Zeng, Y. Q. et al. Electrically pumped topological laser with valley edge modes. *Nature* **578**, 246–250 (2020). 32051601 10.1038/s41586-020-1981-x

[24] Shao, Z. K. et al. A high-performance topological bulk laser based on band-inversion-induced reflection. *Nat. Nanotechnol.* **15**, 67–72 (2020). 31844287 10.1038/s41565-019-0584-x

[25] Zhang, W. X. et al. Low-threshold topological nanolasers based on the second-order corner state. *Light Sci. Appl.* **9**, 109 (2020). 32637076 10.1038/s41377-020-00352-1 PMC7324580

[26] Dikopoltsev, A. et al. Topological insulator vertical-cavity laser array. *Science* **373**, 1514–1517 (2021). 34554782 10.1126/science.abj2232

[27] Yang, L. C. et al. Topological-cavity surface-emitting laser. *Nat. Photonics* **16**, 279–283 (2022).

[28] Contractor, R. et al. Scalable single-mode surface-emitting laser via open-Dirac singularities. *Nature* **608**, 692–698 (2022). 35768016 10.1038/s41586-022-05021-4

[29] Tian, J. Y. et al. Perovskite quantum dot one-dimensional topological laser. *Nat. Commun.* **14**, 1433 (2023). 36918559 10.1038/s41467-023-36963-6 PMC10015034

[30] Wang, Y. et al. Tunable topological lasing of circularly polarized light in a soft-matter-based superlattice. *Laser Photonics Rev.* **17**, 2200643 (2023).

[31] Hwang, M. S. et al. Vortex nanolaser based on a photonic disclination cavity. *Nat. Photonics* **18**, 286–293 (2024).

[32] Leefmans, C. R. et al. Topological temporally mode-locked laser. *Nat. Phys.* **20**, 852–858 (2024).

[33] Li, Z. T., Luo, X. W. & Gu, Q. Topological on-chip lasers. *APL Photonics* **8**, 070901 (2023).

[34] Zhou, Z. C. et al. Prospects and applications of on-chip lasers. *elight* **3**, 1 (2023). 36618904 10.1186/s43593-022-00027-x PMC9810524

[35] Panajotov, K. et al. Vertical-cavity surface-emitting laser emitting circularly polarized light. *Laser Phys. Lett.* **10**, 105003 (2013).

[36] Jia, X. L. et al. Metasurface reflector enables room-temperature circularly polarized emission from VCSEL. *Optica* **10**, 1093–1099 (2023).

[37] Torrelli, V. et al. On-demand polarization by a vertical-cavity surface-emitting laser with two tilted sub-wavelength gratings. *Opt. Lett.* **49**, 3773–3776 (2024). 38950264 10.1364/OL.528268

[38] Pujol-Vila, F. et al. Soft optomechanical systems for sensing, modulation, and actuation. *Adv. Funct. Mater.* **33**, 2213109 (2023).

[39] Xie, M. Y. et al. Flexible multifunctional sensors for wearable and robotic applications. *Adv. Mater. Technol.* **4**, 1800626 (2019).

[40] Pan, J. T. et al. Nonlinear geometric phase coded ferroelectric nematic fluids for nonlinear soft-matter photonics. *Nat. Commun.* **15**, 8732 (2024). 39384797 10.1038/s41467-024-53040-8 PMC11464912

[41] Ryabchun, A. & Bobrovsky, A. Cholesteric liquid crystal materials for tunable diffractive optics. *Adv. Optical Mater.* **6**, 1800335 (2018).

[42] Yang, D. H. et al. Dual-wavelength lasing with orthogonal circular polarizations generated in a single layer of a polymer–cholesteric liquid crystal superstructure. *Polymers* **15**, 1226 (2023). 36904467 10.3390/polym15051226 PMC10007294

[43] Ali, T. et al. A thin-film flexible defect-mode laser. *Adv. Optical Mater.* **8**, 1901891 (2020).

[44] Semenoff, G. W. Condensed-matter simulation of a three-dimensional anomaly. *Phys. Rev. Lett.* **53**, 2449–2452 (1984).

[45] Cayssol, J. Introduction to Dirac materials and topological insulators. *Comptes Rendus Phys.* **14**, 760–778 (2013).

[46] Jung, J. et al. Transport properties of graphene nanoroads in boron nitride sheets. *Nano Lett.* **12**, 2936–2940 (2012). 22524401 10.1021/nl300610w

[47] Dong, J. W. et al. Valley photonic crystals for control of spin and topology. *Nat. Mater.* **16**, 298–302 (2017). 27893722 10.1038/nmat4807

[48] Jiang, J. W., Wang, B. S. & Park, H. S. Topologically protected interface phonons in two-dimensional nanomaterials: hexagonal boron nitride and silicon carbide. *Nanoscale* **10**, 13913–13923 (2018). 29999511 10.1039/c8nr04314k

[49] Xue, H. R., Yang, Y. H. & Zhang, B. L. Topological valley photonics: physics and device applications. *Adv. Photonics Res.* **2**, 2100013 (2021).

[50] He, L. et al. Topologically protected quantum logic gates with valley-hall photonic crystals. *Adv. Mater.* **36**, 2311611 (2024). 10.1002/adma.202311611 38479726

[51] Wehling, T. O., Black-Schaffer, A. M. & Balatsky, A. V. Dirac materials. *Adv. Phys.* **63**, 1–76 (2014).

[52] Huang, S. et al. Interface state in one-dimensional acoustic resonator system with inversion symmetry breaking. *J. Phys. Conf. Ser.* **1828**, 012157 (2021).

[53] Yuan, L. Q. et al. Synthetic dimension in photonics. *Optica* **5**, 1396–1405 (2018).

[54] Ozawa, T. & Price, H. M. Topological quantum matter in synthetic dimensions. *Nat. Rev. Phys.* **1**, 349–357 (2019).

[55] Lustig, E. & Segev, M. Topological photonics in synthetic dimensions. *Adv. Opt. Photonics* **13**, 426–461 (2021).

[56] Wang, Y. et al. Transfer matrix method for light propagation in variable complex chiral media. *Phys. Rev. E* **104**, 064702 (2021). 35030864 10.1103/PhysRevE.104.064702

[57] Fei, H. M. et al. Resonance tunnelling of photons through multiple-well structures in two- dimensional photonic crystals with various well-media. *J. Phys. B: At., Mol. Optical Phys.* **42**, 055401 (2009).

[58] Suthar, B. & Bhargava, A. Pressure sensor based on quantum well-structured photonic crystal. *Silicon* **13**, 1765–1768 (2021).

[59] Jiang, Y., Niu, C. & Lin, D. L. Resonance tunneling through photonic quantum wells. *Phys. Rev. B* **59**, 9981–9986 (1999).

[60] Qiao, F. et al. Photonic quantum-well structures: multiple channeled filtering phenomena. *Appl. Phys. Lett.* **77**, 3698–3700 (2000).

[61] David, A. & Miller, B. Optical physics of quantum wells. In *Quantum dynamics of simple systems* (ed Oppo, G. L.) 239–266 (CRC Press, 1997).

[62] Bernevig, B. A. & Hughes, T. L. 7. Graphene. In *Topological insulators and topological superconductors* (eds Bernevig, B. A. & Hughes, T. L.) 70–90 (Princeton University Press, 2013).

[63] Xiao, D., Yao, W. & Niu, Q. Valley-contrasting physics in graphene: magnetic moment and topological transport. *Phys. Rev. Lett.* **99**, 236809 (2007). 18233399 10.1103/PhysRevLett.99.236809

[64] Xiao, D., Chang, M. C. & Niu, Q. Berry phase effects on electronic properties. *Rev. Mod. Phys.* **82**, 1959–2007 (2010).

[65] Asbóth, J. K., Oroszlány, L. & Pályi, A. *A short course on topological insulators: band structure and edge states in one and two dimensions* (Springer, 2016).

[66] Gao, S. H. et al. Coupling of defect modes in cholesteric liquid crystals separated by isotropic polymeric layers. *Polymers* **10**, 805 (2018). 30960730 10.3390/polym10070805 PMC6403987

[67] Wang, J. Y. et al. Topologically tuned terahertz confinement in a nonlinear photonic chip. *Light Sci. Appl.* **11**, 152 (2022). 35606368 10.1038/s41377-022-00823-7 PMC9126941

[68] Han, C. et al. Lasing at topological edge states in a photonic crystal L3 nanocavity dimer array. *Light Sci. Appl.* **8**, 40 (2019). 31044072 10.1038/s41377-019-0149-7 PMC6478828

[69] Zhang, T. C. et al. Twisted moiré photonic crystal enabled optical vortex generation through bound states in the continuum. *Nat. Commun.* **14**, 6014 (2023). 37758708 10.1038/s41467-023-41068-1 PMC10533549

