# Supplementary material for: Soft-matter-based topological vertical cavity surface emitting lasers
Source: Light Sci Appl. 2026 Jan 2;15:27. doi: 10.1038/s41377-025-02011-9 (PMC12757594; doi:10.1038/s41377-025-02011-9)
Supplement: Supplementary file 1 — Supporting Information [file 41377_2025_2011_MOESM1_ESM.docx]

Supplementary Information for

**Soft-Matter-Based Topological Vertical Cavity Surface Emitting Lasers**

Yu Wang^1,*^, Shiqi Xia^1,*^, Qun Xie^1^, Donghao Yang^1^, Jingbin Shao^1^, Xinzheng Zhang^1, 2, 3, ‡^, Irena Drevensek-Olenik^4^, Qiang Wu^1^, Zhigang Chen^1, 2, ‡^ and Jingjun Xu^1, ‡^

*1* The MOE Key Laboratory of Weak-Light Nonlinear Photonics*,* TEDA Institute of Applied Physics and School of Physics, Nankai University, Tianjin, 300457, China

*2* Collaborative Innovation Center of Extreme Optics, Shanxi University, Taiyuan, Shanxi 030006, China

*3* International Sino-Slovenian Joint Research Center on Liquid Crystal Photonics, Nankai University, Tianjin, 300071, China

*4* Faculty of Mathematics and Physics, University of Ljubljana, and Department of Complex Matter, J. Stefan Institute, SI-1000 Ljubljana, Slovenia

* Authors contributed equally to this article.

‡ Corresponding authors: zxz@nankai.edu.cn; zgchen@nankai.edu.cn; jjxu@nankai.edu.cn

**Supporting Note 1.** **Quantum size effect in a single optical quantum well**

Generally, an optical quantum well can be formed by inserting a photonic crystal or a uniform dielectric material into another photonic crystal with a different energy band structure. Here, we take advantage of the one-dimensional (1D) photonic crystal properties of polymerized cholesteric liquid crystals (PCLCs), whose photonic bandgap can be regarded as a potential barrier. As a kind of bestselling polyester film for its versatile and reliable performance, isotropic Mylar layers inserted into PCLCs can be regarded not only as the defect layers but also as potential wells. Thus, PCLCs together with Mylar layers form a metastructural superlattice. This structure has many advantages, since the thicknesses of each barrier and potential well can be designed and changed readily according to the requirements, so that the quantized energy states and density distribution in the optical superlattice can be changed. As a result, it can flexibly adjust the resonant frequencies, bandwidth and optical modes of the microcavity.

Due to the thin Mylar layer acting as a potential well, the motions of photons appear quantized characteristics and their energies take discrete values. That is, photons of different energies occupy different discrete energy levels, forming energy eigenstates in the form of standing waves, or quantum well states. As shown in Fig. S1, by changing the thickness of the Mylar film, one can change the width *x* of the potential well, so do the energies and the spacing of energy levels within the potential well. When the width of the well becomes wider, the energy corresponding to the *k*^th^ energy level gets lower and the spacing between adjacent energy levels becomes narrower. With the increase of *x,* the resonant wavelength corresponding to the same order has a redshift, as shown by arrows with the same color in the right column of Fig. S1. Table S1 shows how the resonant wavelengths of the energy levels belonging to the same order change with the width of the potential well, when there are only two energy levels inside the well. In addition, it is noteworthy that when the thickness of the Mylar film increases from 3 μm to 3.165 μm, the (*k*+1)^th^ level appears in the potential well (3.165 μm), and its energy coincides with the *k*^th^ energy level of the former potential well (3 μm). This means that if we choose the 3 μm- and 3.165 μm-thick Mylar films as A and B sites to form a diatomic chain structure, Dirac point will also appear in the structure that breaks the inversion symmetry, which is an extension of the traditional two-level systems. This is caused by the fact that the optical quantum well system has multiple quantized energy levels.


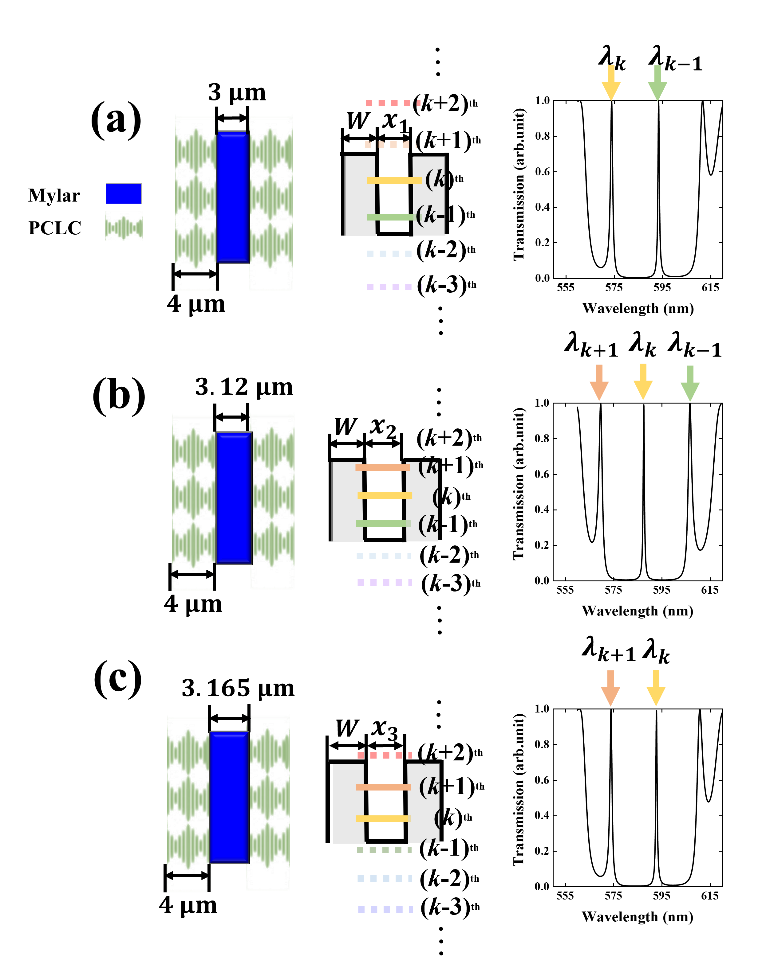


**Fig. S1. Quantum size effect in a single optical quantum well**. (a-c) Schematic diagrams of the structures consisting of a single-layer Mylar film sandwiched in two PCLC layers (left column), diagrams of the single potential well corresponding to the three-layer structures (middle column) and their transmission spectra (right column). The thicknesses of the Mylar films in (a-c) are 3 μm, 3.12 μm and 3.165 μm, respectively.

**Table S1. Resonant wavelengths** $\boldsymbol{\lambda}$ **of energy levels belonging to *the same order* for different widths *x* of the potential wells**

| ***x* (**$\boldsymbol{\mu m}$**)** | $\boldsymbol{\lambda}$ **(nm)** | |
| --- | --- | --- |
| **3** | **574.0** | **593.5** |
| **3.02** | **576.2** | **595.9** |
| **3.04** | **578.4** | **598.2** |
| **3.06** | **580.6** | **600.5** |
| **3.08** | **582.9** | **602.7** |
| **3.10** | **585.3** | **604.8** |
| **3.12** | **587.6** | **606.7** |

**Supporting Note 2.** **Band crossing point in the** **metastructural superlattice with different potential wells A and B**

As shown in Table S2, for different Mylar films with expressly designed thicknesses, optical quantum wells can always have an energy level with the same energy corresponding to the wavelength of 574 nm, although they belong to different orders. This means that if any two of them are selected as potential wells A and B to form the binary optical superlattice, energy levels with equal energy will couple and split to form mini-bands, resulting in the closure of the mini-band gap at this energy.

For example, as shown in Fig. S2a, potential wells A and B in the superlattice are Mylar films with thicknesses of 3 μm and 3.165 μm, respectively, and the PCLC films with the same thickness of 4 μm act as potential barriers. If we adopt energy levels corresponding to the wavelength of 574 nm as the on-site potentials of A and B sites, and also define *M* to describe the energy difference between the on-site potentials, we can find *M* = 0 in this superlattice, so that the band gap closes. In this case, the mini-band gaps of the two sub-structures in the gray and red areas are closed at 574 nm due to the coupling of the (*k*+1)^th^ energy level in the potential wells B and the *k*^th^ energy level in the potential wells A, as shown in the Fig. S2b-e. As a result, there is no topological interface state (TIS) in this mini-band gap after juxtaposition of these two different structures with the same topological properties, as shown in the Fig. S2f, h. Besides, the distributions of eight eigenstates corresponding to the resonant wavelengths are shown in Fig. S2g. Obviously, there is no TIS localized in the structure.

**Table S2. Resonant wavelengths** $\boldsymbol{\lambda}$ **of energy levels belonging to *different orders* for different widths *x* of the potential wells**

| ***x* (**$\boldsymbol{\mu m}$**)** | $\boldsymbol{\lambda}$ **(nm)** | |
| --- | --- | --- |
| **3** | **574** | **593.5** |
| **3.165** | **574** | **592.9** |
| **3.33** | **574** | **592.3** |
| **3.495** | **574** | **591.7** |
| **3.66** | **574** | **591.2** |
| **3.825** | **574** | **590.6** |
| **3.99** | **574** | **590.2** |


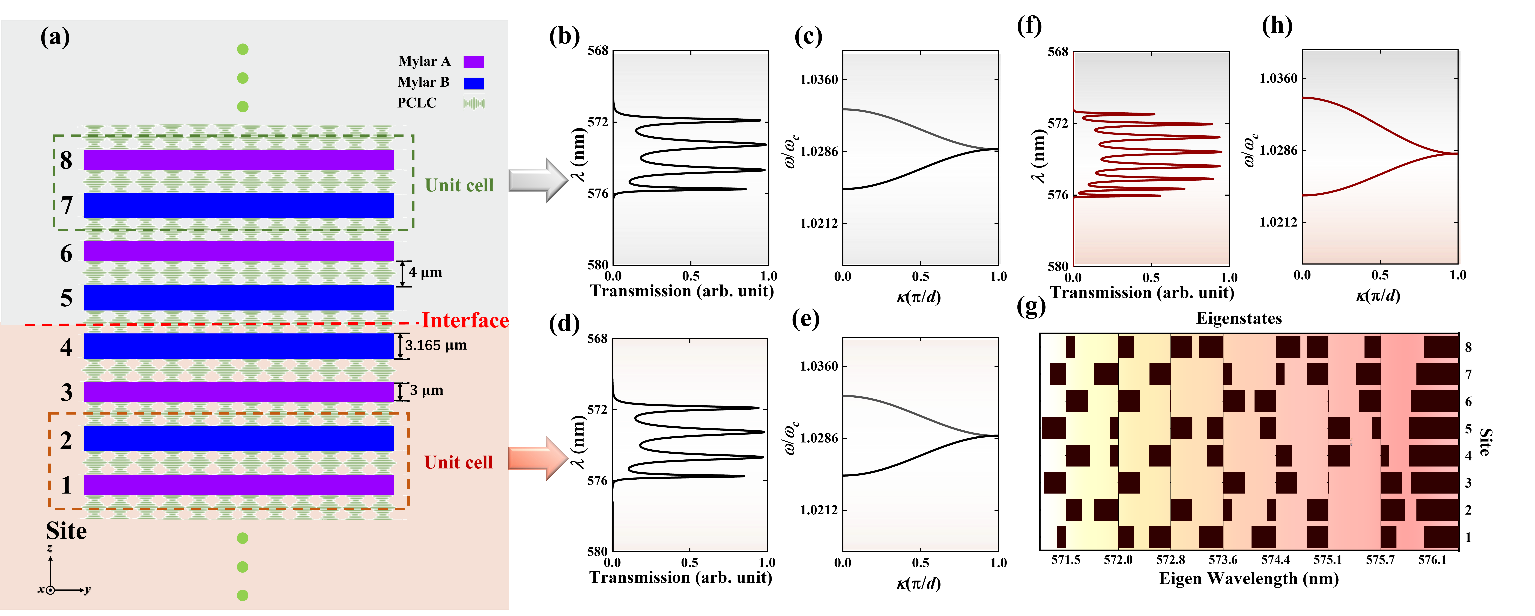


**Fig. S2. Band crossing point in the** **binary optical superlattice with different potential wells A and B.** (a) Diagram of the juxtaposed optical superlattice, in which gray and red areas are corresponding to different superlattices with same topological invariants. (b, d) Transmission spectra and (c, e) the mini-band structures of the superlattices in the gray and red regions, respectively. (f) Transmission spectrum and (h) the mini-band structure of the juxtaposed optical superlattice. (g) Distributions of eigenstates corresponding to eight eigen-wavelengths.

**Supporting Note 3.** **Band inversion process in the superlattices with asymmetric double optical quantum wells**

Here, we choose a 3$\text{ }\text{μm}$ -thick Mylar film A and a 4$\text{μm }$-thick Mylar film B to construct two superlattices with different topological properties by exchanging Mylar films A and B, as shown in Fig. S3a, d. The unit cells of the structures can be regard as asymmetric double optical quantum wells. Due to resonant tunneling effect, the original degenerate energy levels split and cause a series of splitting resonant peaks in the transmission spectra, as shown in Fig. S3b, e. In turn, mini-bands are formed and mini-band gaps are generated, as shown in Fig. S3c, f. This phenomenon caused by resonant tunneling in optical quantum wells can also be analyzed according to the tight binding approximation. For two adjacent asymmetric potential wells, only when the energy levels belonging to the same order or different orders have similar energies, can they couple to each other and cause the resonant tunneling and quantum interference of the transmitted photons. In addition, as shown by insets in Fig. S3c, f, the electric field distributions of the eigenstates at the edge of the first Brillouin regions marked by K, L, M, N have opposite symmetries, which indicates that the upper and lower bands next to the common band gap of two structures undergo a band inversion process.

**
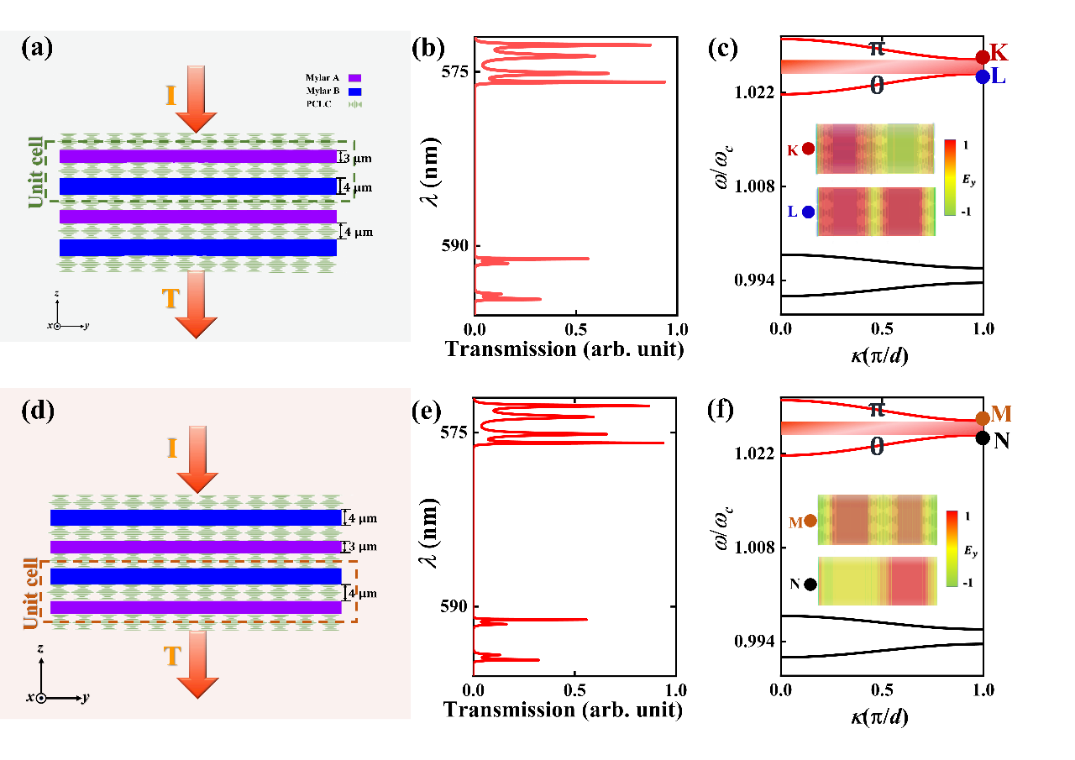
**

**Fig. S3. Band inversion process in the inversion symmetry broken optical superlattices.** (a, d) 1D optical superlattices consisting of PCLCs and two kinds of Mylar films. I: Incident. T: Transmission. (b, e) Transmission spectra, (c, f) mini-band structures corresponding to optical superlattices shown in (a) and (d), respectively. Insets show the electric field distributions of the eigenstates at the edges of the first Brillouin regions marked by K, L, M, N within a unit cell.

**Supporting Note 4. Transmission spectra of the topological VCSEL**


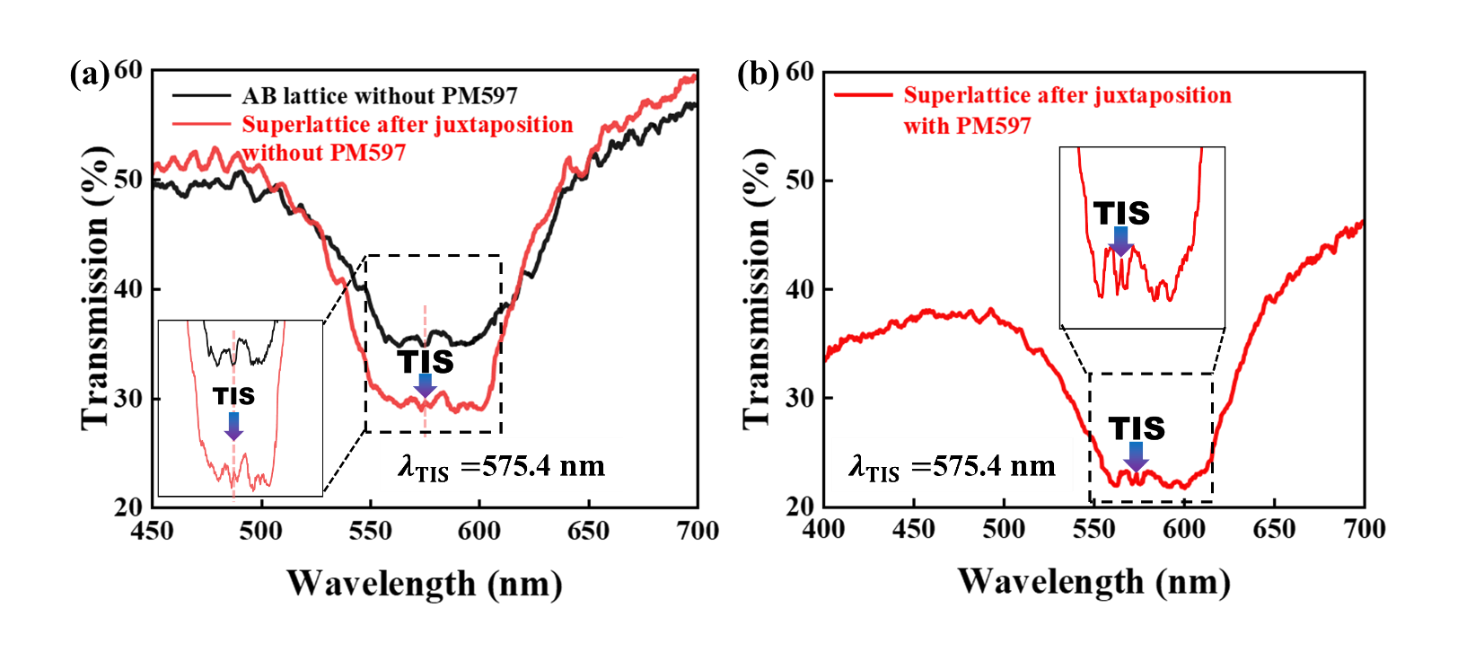


**Fig. S4. Experimentally measured transmission characteristics of a 1D superlattice and a topological VCSEL.** (a) Transmission spectra of the AB lattice (black) before juxtaposition and (red) the juxtaposed superlattice without dye PM597. (b) Transmission spectrum of the juxtaposed superlattice with dye PM597. The schematic diagram of the juxtaposed superlattice structure is shown in Fig. 3a of the main text. The insets display a magnified view of the band gaps.

As shown in Fig. S4, we measured the transmission characteristics of the AB superlattice before juxtaposition, and the juxtaposed superlattices in Fig. 3a with and without dye PM597. A deuterium halide natural white light source (DH-2000-BAL, Ocean Optics) was used to irradiate the sample through a lens with a focal length of 85 mm for measuring the transmission spectrum. The transmitted light, after passing through the sample, was collected by another lens with the same focal length and coupled to a spectrometer (HR4000CG-UV-NIR, Ocean Optics). However, due to the selective reflection and unavoidable light scattering of the PCLC films, the transmittance of the stacked multilayer structure will be greatly reduced. Therefore, the multilayer structures formed by PCLC films in our current experimental studies have a limited number of layers. Also limited by the resolution of the spectrometer, we could not observe sharp transmission peaks for the time being, but we could still find several transmission peaks corresponding to the TIS with wavelength of 575.4 nm and to other bulk resonant modes in the transmission spectra of the juxtaposed superlattices with and without dye PM597. From Fig. S4a, we can clearly see that the TIS is located in the center of the mini-band gap. These results are in good agreement with our theoretical calculations, as shown in Fig. 3c. Since the band gap of the PCLC we used ranges from 560 nm to 604 nm, the samples reflect yellow light.

**Supporting Note 5.** **Eigen wavelengths and the inverse participation rates of the bulk sates and the TIS under increasing disorder**

Here, we study the influence of on-site potential disorder ($E_{i}+\delta_{i}$) on the energy eigenvalues and localization of the bulk states and the TIS. The model structure is the same as that in Fig. 3a of the main text, but we choose the total number of potential wells in the superlattice as 7200, approximately to an infinite system in the theoretical calculation. We limit the disorder disturbances on the on-site potential to be less than 0.5%, and without considering disturbance to the coupling strength. This requirement can be expressed as $\delta_{i}=0.5\%\gamma\zeta_{i}$, where$\gamma\in[0,1]$ is a disturbance factor that determines the overall degree of disorder. $\zeta_{i}$ is a random number between -1 and +1, where *i*=1, 2, ..., 7200 represents the site index in the superlattice. Since the TIS is particularly different from other bulk states and is highly localized at the layers on both sides of the juxtaposing interface, the inverse participation rate (IPR) can be introduced to describe the localization degree of each eigenstate, which is defined as:

$\mathrm{IPR}^{(n)}=\frac{\sum_{i} {|\psi_{i}^{(n)}|}^{4}}{{(\sum_{i} {|\psi_{i}^{(n)}|}^{2})}^{2}}$ (1)

Here, $\psi_{i}^{(n)}$ is the eigenstate corresponding to the *n*^th^ eigen energy. Due to the large size of the system in this section, the IPR of the highly localized TIS should be 1, while those of the bulk states are close to 0 without disturbance. When the disordered disturbance$\delta_{i}$ is applied to all diagonal elements in the Hamiltonian matrix, and 50 groups of calculations are carried out. Fig. S5a, b illustrate one group of the calculated results on the change of the eigen-wavelengths and IPR of 7200 eigenstates with different disorder degree of the on-site potentials. Different colors in the figures represent different disorder degrees. With the increase of the disorder factor $\gamma$, the eigen-wavelengths corresponding to the TIS in the center of the band gap and to the bulk states all gradually deviate from their original positions, while the shift of the bulk states is larger than that of the TIS. In addition, the IPRs of the bulk states change greatly, while that of the TIS remains unchanged. For quantitative description, we choose one set of the bulk states (in the red dotted boxes of Fig. S5a, b) and one set of the TISs (in the black dotted boxes of Fig. S5a, b) to show the shift of the eigen wavelengths and the change of the IPRs with the degree of disorder in Fig. S5c, d. The standard deviations of 50 groups of calculated results are shown as error bars in the figures. Obviously, the TIS can basically keep its corresponding eigen-wavelength unchanged until $\gamma$ is equal to about 0.25. However, the localization of the TIS is not affected with the increasing degree of disorder, which is always highly localized. The bulk state is affected upon the introduction of the disordered disturbance. Therefore, compared with bulk states, the TIS is more robust against the disorder disturbance on the on-site potentials.

Given that the fabrication and stacking processes of our multi-layer thin-film topological VCSEL inherently introduce minor thickness and flatness irregularities, these imperfections inevitably act as disorder perturbations in on-site potentials and coupling coefficients within the structure. To verify the robustness of the TIS lasing experimentally, we performed pumping at different positions on the same sample and used a spectrometer equipped with a fine grating (resolution: 0.05 nm) to improve measurement accuracy. We observed that the wavelength shift remained within 0.7 nm across all measured positions (see Fig. S5e). The constant appearance of the TIS indicates that it is robust to the disorder and imperfections in experimental processing.

**
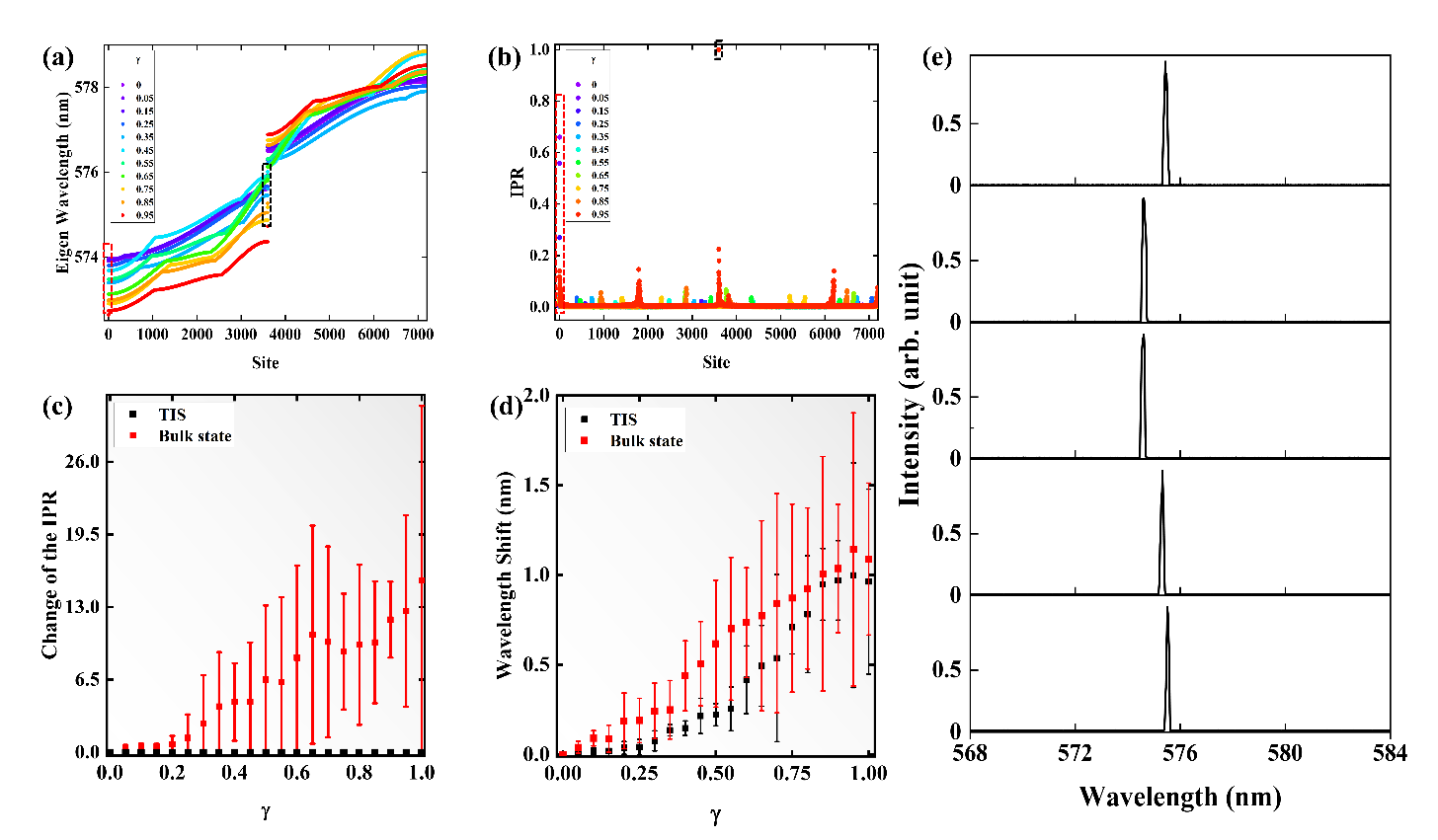
**

**Fig. S5. Bulk states and the topological interface state under increasing disorder.** (a) Calculated eigen-wavelengths and (b) IPR of the bulk sates and the TIS under increasing disorder. Different colors represent different degree of disorder *γ,* as shown in the legend*.* (c) Shift of the eigen-wavelengths and (d) change of the IPR corresponding to the TIS and one bulk state with the degree of disorder *γ*. (e) Experimentally measured emission spectra at different locations of the same sample.

**Supporting Note 6.** **Divergence angle of the topological VCSEL**

We measured the beam divergence angle of the laser using a beam profile analyzer capable of capturing the spot intensity distribution and enabling spot size calculations. When the pump energy was lower than the threshold, we found the fluorescence was emitted in all direction. For the measurement, the detector was positioned at the focal point of a thin lens with a 13 mm focal length and in the direction perpendicular to the surface of the sample. The divergence angle of the light beam can be obtained as $\text{θ=}\frac{\text{w}_{\text{F}}}{\text{f}}$, where *w*_F_ is the spot size at the focal plane of the lens measured by the beam profile analyzer (the diameter at the peak intensity of 1/e²) and *f* is the focal length of the lens. From the spot size in Fig. S6, the divergence angles measured at pump energy densities of 3.8 mJ$\cdot$cm^-2^ (below threshold) and 12.74 mJ$\cdot$cm^-2^ (above threshold) are 17° and 3°, respectively. It is worth noting that since the fluorescence before the threshold is a divergent spherical wave, the divergence angle we measured is limited by the numerical aperture of the lens, and the actual fluorescence divergence angle will be greater than this value. According to the measurements, our topological VCSEL achieves directional emission.


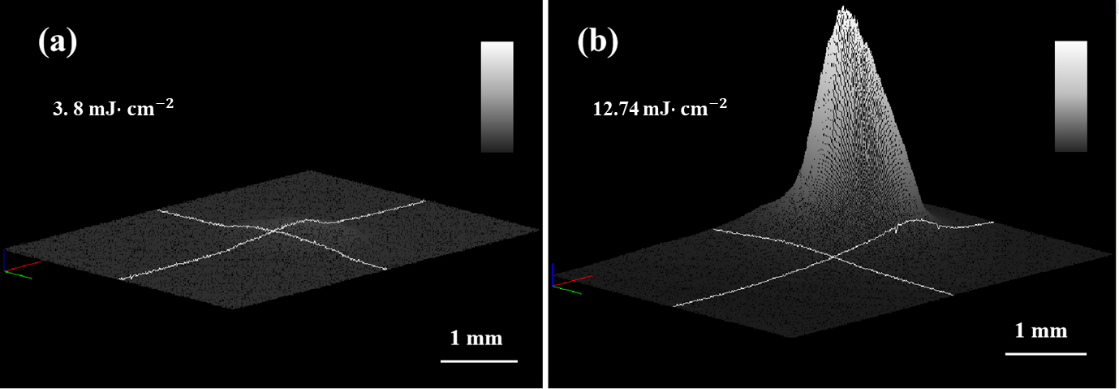


**Fig. S6.** **Experimentally measured beam profiles in the direction perpendicular to the surface of the sample (a) before and (b) after the lasing threshold.** The divergence angle of the emission beam calculated at 3.8 mJ$\cdot$cm^-2^ was 17°, while decreased to 3° of the lasing beam at 12.74 mJ$\cdot$cm^-2^.

**Supporting Note 7.** **Theoretical simulations of the topological VCSEL**

In order to further verify the topological characteristics of the lasing, we conducted theoretical simulations of the superlattice structure (Fig. S7a) used for the topological VCSEL by using the FDTD Solutions software. To simulate the emission spectrum, we added an imaginary part to the refractive index of the Mylar film layer in the superlattice structure. By placing the pump source at one side of the structure and setting up two monitors respectively at the other side of the structure and along the *z*-direction, the calculated emission spectrum and spatial distribution were obtained. The wavelength of topological lasing in the calculated emission spectrum shown in Fig. S7b corresponds well to the experimental result (Fig. 5a in the main text). Meanwhile, the calculated spatial distribution shows that the light intensity is mainly localized at the interface, as shown in Fig. S7c. This result also has a good consistency with the eigenstate distribution of the TIS calculated through the tight-binding model (Fig. 2b and Fig. 3b in the main text), proving that this lasing originates from the TIS.


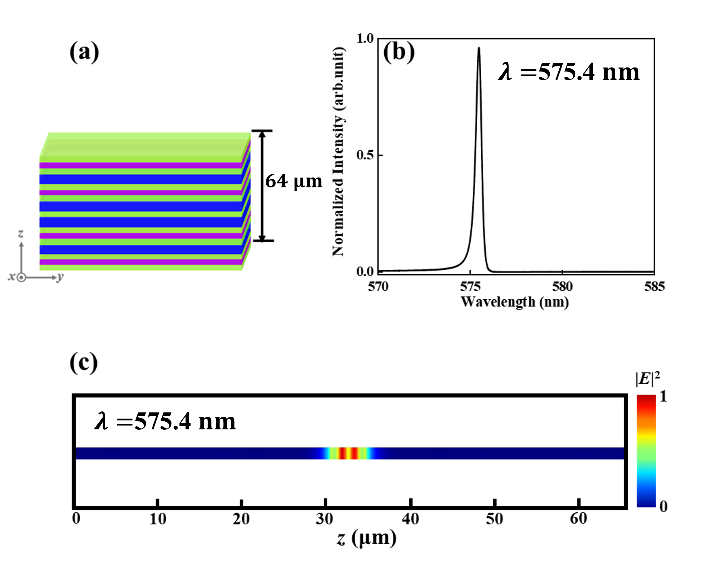


**Fig. S7. Theoretical simulations of the topological VCSEL.** (a) Superlattice structure of the topological VCSEL used in the simulations. (b) Calculated emission spectrum. (c) Calculated spatial distribution of the TIS along *z* direction.

**Supporting Note 8.** **Stability of the topological VCSEL**

In order to verify the long-term stability of our topological VCSEL device, we tested the emission spectrum again after fourteen months of storage and many times of bending. The emission spectra measured 14 months ago and the one measured recently are shown by the red and black curves, respectively. The experimental conditions were set the same, at a pump energy of 1.25 μJ and using the same experimental set up. Here, the emission spectra look a little different from that in the main text, which is due to that part of the fluorescence was collected when the objective was placed only 0.5 cm behind the sample in these two tests. As shown in Fig. S8, the topological VCSEL still maintains its original laser characteristics, including the lasing wavelength as well as the linewidth, while only the signal-to-background ratio is slightly reduced.

**Fig. S8. Direct comparison of emission spectra showing long-term stability of the sample used for our topological VCSEL.** Emission spectra measured fourteen months ago (red curve) and currently (after fourteen months of storage and many times of bending) (black curve).

**Supporting Note 9.** **Fluorescence spectrum of the gain medium**

Among commonly used fluorescent dyes, Pyrromethene 597 (PM597) uniquely satisfies the absorption requirements at 532 nm, substantial Stokes shift, strong emission at 575.4 nm, and favorable physicochemical properties, which make it the optimal choice for our gain medium. Here, we measured the fluorescence spectrum of 1.0 wt.% PM597 solution in ethanol, whose fluorescent band ranges from 535 nm to 615 nm, as shown in Fig. S9.


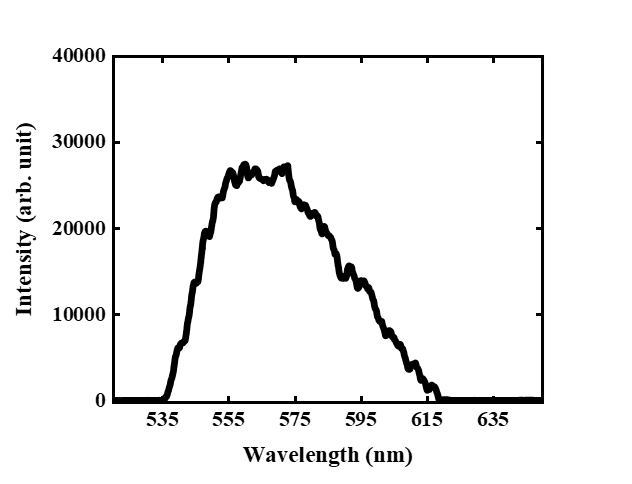


**Fig. S9. Fluorescence spectrum of 1.0 wt.% PM597 solution in ethanol.**
